# Chemical Characterization, Antioxidant, Enzyme Inhibition and Antimutagenic Properties of Eight Mushroom Species: A Comparative Study

**DOI:** 10.3390/jof6030166

**Published:** 2020-09-09

**Authors:** Sinan Alkan, Ahmet Uysal, Giyasettin Kasik, Sanja Vlaisavljevic, Sanja Berežni, Gokhan Zengin

**Affiliations:** 1 Organic Agriculture Administration Department, Çumra School of Applied Sciences, Selcuk University, Çumra, 42250 Konya, Turkey; sinanalkan42@gmail.com; 2 Department of Medicinal Laboratory, Vocational School of Health Services, Selcuk University, 42130 Konya, Turkey; ahuysal@selcuk.edu.tr; 3 Department of Biology, Science Faculty, Selcuk University, 42130 Konya, Turkey; giyasettin@selcuk.edu.tr or; 4 Department of Chemistry, Biochemistry and Environmental Protection, Faculty of Sciences, University of Novi Sad, Trg Dositeja Obradovica 3, 21000 Novi Sad, Serbia; sanja.vlaisavljevic@dh.uns.ac.rs (S.V.); sanja.beric@dh.uns.ac.rs (S.B.)

**Keywords:** mushrooms, bioactive compounds, antioxidants, enzyme, pharmaceuticals

## Abstract

This study aimed to determine the chemical composition and biologic activities of eight mushroom species (*Amanita crocea*, *Hemileccinum depilatum*, *Cyclocybe cylindracea*, *Lactarius deliciosus*, *Hygrocybe acutoconica*, *Neoboletus erythropus*, *Russula aurea* and *Russula sanguinea*). The antioxidant, enzyme inhibitory and mutagenic/antimutagenic activities were evaluated to provide data on the biologic activities. With respect to the chemical composition, LC–MS/MS technique was used to determine individual phenolic compounds present in the extracts. Antioxidant properties were investigated by different chemical methods including radical quenching (DPPH and ABTS), reducing power (CUPRAC and FRAP), phosphomolybdenum and metal chelating. In the enzyme inhibitory assays, cholinesterases, tyrosinase, amylase and glucosidase were used. Mutagenic and antimutagenic properties were evaluated by the Ames assay. In general, the best antioxidant abilities were observed from H. depilatum and N. erythropus, which also showed highest level of phenolics. The best cholinesterase inhibition ability was found from C. cylindracea (1.02 mg GALAE/g for AChE; 0.99 mg GALAE/g for BChE). Tyrosinase inhibition ability varied from 48.83 to 54.18 mg KAE/g. The extracts exhibited no mutagenic effects and showed significant antimutagenic potential. *H. acutoconica*, in particular depicted excellent antimutagenicity with a ratio of 97% for TA100 and with a rate of 96% for TA98 strain against mutagens in the presence of metabolic activation system. Results presented in this study tend to show that the mushroom species could be exploited as potential sources of therapeutic bioactive agents, geared towards the management of oxidative stress, global health problems and cancer.

## 1. Introduction

In this century, it is anticipated that the world population will exceed nine billion, and hence human beings will face with several challenges. Health and food security will be the top of these problems. The scientific community have scrutinized plants and mushrooms to solve these two problems. In particular, mushrooms have attracted much interest as they have less fats and calories, but higher protein and vitamins [1]. Therefore, nutritious foods represent a vital component of food safety, which means that people have a balanced nutritional profile and a sufficient intake is essential to maintain a healthy lifestyle [2]. In the published literature, a number of studies have focused on the medical benefits associated with mushroom consumption, particularly against chronic and degenerative diseases, including the treatment of obesity and cardiovascular disorders, [3]. Numerous mushrooms have been reported to be edible and at the same time offer medicinal properties, which can be exploited as nutraceuticals and/or functional foods [4,5,6].

A mushroom is defined as a macro mushroom with a different fruit body, which can be hypogeous, epigeous or on plants, large enough to be visible to the naked eye and to be collected by hand (Chang & Miles, 2004). Wild mushrooms are gaining worldwide popularity in recent years, recognizing the fact that they are a good source of delicious food with high nutritional value [7]. The systematic studies to identify mushroom species in Turkey is continuing rapidly by scientists. Through systematic studies which began in the 1850s in Turkey, today the number macrofungi species have exceeded 2200 approximately based on recent systematic studies [8,9,10,11,12,13].

Taking into consideration the above-mentioned information, this study was designed to determine the biologic properties and chemical profiles of eight mushroom species (*Amanita crocea*, *Hemileccinum depilatum*, *Cyclocybe cylindracea*, *Lactarius deliciosus*, *Hygrocybe acutoconica*, *Neoboletus erythropus*, *Russula aurea* and *Russula sanguinea*) from Turkey. The mushrooms species were collected from same specific area, whereby most of the species edible by local people form that region. However, only one mushroom (*H. acutonica*) is not edible (Table 1). Biologic properties included the antioxidant, enzyme inhibitory and mutagenic/antimutagenic effects. Chemical profiles were also established by LC–MS/MS technique. It is anticipated that results generated from study, could be a cornerstone for designing further studies on these mushroom species.

## 2. Materials and Methods

### 2.1. Mushrooms Material and Preparation of Extracts

The mushroom materials were collected from Konya (Beykonağı village, Ilgın) in 2018. The mushroom samples (about 10 fruiting bodies) were identified by the mycologists Dr. Sinan Alkan and Dr. Giyasettin Kasik (Table 1). The mushroom species were identified by both morphologic and microscopic (spore properties) observations. The fresh mushroom materials were carefully cleaned using a plastic knife. The mushroom samples were then placed in snap-lock plastic bags and frozen at −80 °C. Five fruiting bodies for each species were used to obtain the extracts. Other were stored in the fungarium at Selcuk University, Konya. Five fruiting bodies for each species were dried in an oven (48 h, 40 °C). After drying process, the samples were powdered using a laboratory mill.

To prepare the extracts, maceration technique was used. The powdered mushroom materials (5 g) were macerated with 100 mL of methanol for 24 h at room temperature. Then, the mixture was filtered and evaporated using a rotary evaporator. All extracts were stored at 4 °C until analysis.

### 2.2. Assay for Total Phenolic Content

The total phenolic contents of the mushroom extracts were measured as reported in previous publications [14,15]. Gallic acid (GAE) was used to quantify the levels of total phenolics.

### 2.3. LC–MS/MS Quantification of Phenolic Compounds

Lyophilized extracts were dissolved in water to obtain 10 mg/mL (stock solution) and stored at −4 °C. The chemical profiles were conducted by [16] and 45 standard compounds were used. Data were evaluated by dynamic MRM (retention time, precursor ion, product ion, voltage of fragmentor, collision voltage). For all the compounds, peak areas were calculated using Agilent MassHunter Workstation software—Qualitative Analysis (ver. B.03.01.). Calibration curves were plotted, and samples’ concentrations calculated using the OriginLabs Origin Pro (ver. 9.0) software.

### 2.4. Determination of Antioxidant and Enzyme Inhibitory Effects

Reducing power, metal chelating, phosphomolybdenum and free radical scavenging assays were preferred as antioxidant assays. Standard compounds including trolox (TE) and EDTA (EDTAE) were used to express antioxidant abilities. Enzyme inhibitory abilities were tested against different enzymes including α-glucosidase, α-amylase, cholinesterases and tyrosinase. Standard inhibitors namely acarbose, kojic acid and galantamine were used to express the enzyme inhibition abilities. The details for these assays were given in our earlier study Grochowski et al. [17]. All details are also given in Supplementary Materials. 

### 2.5. Antimutagenic/Mutagenic Properties

In the present study, mutagenic potential of eighth mushrooms were evaluated by Salmonella/microsome test system in the presence and absence of metabolic activation at the same time. The plaque incorporation method was employed with two mutant Salmonella strains. *Salmonella typhimurium* TA98 strain was capable for detecting of frame shift mutations while *S. typhimurium* TA100 strain was competent for elucidating of base pair exchange mutations. These two strains were kindly obtained from Microbiology Research Laboratory, Vocational School of Health Services, Selcuk University and are maintained as described by Maron and Ames [18]. The mushroom extracts were subjected to toxicity testing described by Dean et al. [19]. Hence, nontoxic dose levels of the extracts were revealed. According to the results of the test it was determined that 10,000 µg/plate and lower doses manifested no toxicity both for two strains of Salmonella with and without S9 mix.

The Ames test was employed as described below: The overnight cultures (16 h) of test strains were obtained in an orbital shaker and adjusted to turbidity of 1–2 × 10^9^ cfu/mL. Then, 100 µL of fresh bacteria culture, 500 µL Na–P buffer (0.2 M, pH 7.4 for experiment without S9 mix) and 100 µL different extract concentrations were added to top agar (2.5 mL) supplemented with (0.5-mM L-histidine/D-biotin solution) and gently mixed by vortex. Then complete mixture was poured on minimal agar plates. The plates were incubated at 37 °C for 48–72 h and the revertant bacterial colonies of each plate were counted. Data were collected with a mean ± standard deviation of two assays (*n* = 3). In each experiment positive and negative controls were included routinely. The potential mutagenicity was considered for extracts when a dose–response relationship was detected and a two-fold increase in the number of revertants was observed for at least one concentration [18].

For the antimutagenic evaluation of mushroom extracts, plate incorporation method described by Maron and Ames [18] was conducted with some modifications [20]. Three different doses of mushroom extracts were treated with positive mutagens both in the presence and absence of metabolic activation. A hundred µL of fresh bacterial culture, 500 µL of metabolic activation enzymes (S9 mix) (or Na–P buffer for the assay without S9), 100 µL of known mutagen solutions and 100 µL mushroom extracts were added to 2.5 mL top agar (at 45 °C). Vortexed mixture was poured onto the MGA plates and after solidification all plates were incubated 48–72 h at 37 °C and the number of revertant colonies per plate was counted. For each assay triplicate plates were used. After incubation period the revertant colonies were counted and plates including only positive mutagen (without mushroom extract) were determined as 0% inhibition. The antimutagenic potential (Inhibition) was determined by equation: [(A − B)/(A − C)] × 100, where A = No. of his. revertants in the absence of sample, B = No. of his. revertants in the presence of sample, C = spontaneous revertants [20]. If the inhibition rates were 25% or lower, it was defined as no antimutagenicity or weak activity; 26–39% rates were defined as moderate or temperate activity; 40% and higher inhibition rates were determined as strong antimutagenicity [21].

### 2.6. Data Analysis

All assays were performed in triplicate. The results were expressed as mean ± standard deviation (SD). One-way analysis of variance (ANOVA) followed by Tukey’s post hoc test with *α* = 0.05 were conducted under Xlstat software environment version 2018. Afterwards, principal component and hierarchical clustering analyses were carried out with R software v.3.6.1 by using the package FactoMineR.

## 3. Results and Discussion

### 3.1. Chemical Composition

Phenolic compounds contain one or more hydroxyl groups in the aromatic rings, and they exhibit a broad spectrum of biologic abilities including antioxidant, antimicrobial and anti-inflammatory. Thus, these compounds are gaining a great interest in pharmaceutical and food areas [22,23,24]. From this perspective, we investigated total phenolic content of the mushroom extracts and the results are given in Table 2. It was observed that *H. depilatum* contained the highest level of phenolic (20.10 mg GAE/g extract), followed by *N. erythropus* (16.52 mg GAE/g) and *R. aurea* (11.36 mg GAE/g). The lowest content was also determined in *L. deliciosus* with the value of 7.42 mg GAE/g. In literature, different level of phenolics in the mushroom samples were observed [7,25,26,27]. The differences levels could be explained by several factors such as different geographical locations and collection seasons. As an another subject, the spectrophotometric Folin–Ciocâlteu assay could not reflect accurate levels of phenolics in the plants or mushrooms extracts because the reagent could be also react with peptides and other chemicals [28]. To this end, at least one chromatographic method such as HPLC, LC–MS or GC–MS need to confirm the precise level of total bioactive compounds. In the current study, the chemical profiles of the mushroom extracts were investigated by LC–MS/MS. As can be seen in Table 3, the mushrooms contained very low level of phenolics and hinic acid was determined in all mushroom extracts. *p-* hydroxybenzoic acid was another important compound and the highest level of it was determined in *C. cylindracea* extract (105.73 µg/g extract). Interestingly, cinnamic acid was only determined in *A. crocea* (539.38 µg/g extract). In addition, chlorogenic acid was determined in *C. cylindracea* (6.81 µg/g extract) and *L. deliciosus* (5.49 µg/g extract). Matairesinol (7.68 µg/g extract) and amentoflavone (1.02 µg/g extract) were just determined in *N. erythropus*.

### 3.2. Antioxidant Effects

Oxidative stress, which is an imbalance between free radicals and antioxidant, is closely linked to several health problems including Alzheimer’s disease, diabetes mellitus and cardiovascular diseases. In this respect, several novel and effective antioxidants relevant in the management and prevention of the diseases are being probed [29,30,31]. Thus, the antioxidant propensities of the mushroom extracts were investigated via several assays including free-radical quenching, reducing power, metal chelating and phosphomolybdenum. The results are given in Table 2. DPPH and ABTS are the most popular radicals in the in vitro antioxidant assays to evaluate radical scavenging ability. In these assays, *H. depilatum* exhibited the best activity (84.33 mg TE/g for ABTS and 41.89 mg TE/g for DPPH); (N) *erythropus* showed the second highest radical quenching ability, while the weakest ability was observed by *L. deliciosus*. FRAP and CUPRAC are reducing power assays, which contain the reduction of metals (from Fe^3+^ to Fe^2+^ in FRAP assay and from Cu^2+^ and Cu^+^ in CUPRAC assay). Similar to radical scavenging assays, *H. depilatum* and *N. erythropus* depicted the strongest reductive abilities in these assays. These mushrooms had also the highest level of total phenolics. In this context, the observed abilities may be explained by the presence of phenolics. This fact was also confirmed by several authors [32,33,34], who reported a strong correlation between total phenolics and antioxidant properties of some mushroom extracts. Transition metals play significant role in the production of hydroxyl radical via Fenton reaction and thus, the chelation of them is one important antioxidant mechanism. As can be seen in Table 2, the metal chelating ability of the tested mushrooms were very close. The best chelating ability was recorded for *A. crocea* with the value of 14.91 mg EDTAE/g, while *N. erythropus* had the lowest chelating ability. When compared with other antioxidant assays, the results were different in the metal chelating ability. These contradictory results may be explained by the presence of non-phenolic chelators such as peptides or vitamin C. In addition, some authors reported that the metal chelation ability of phenolic is a minor way in their antioxidant mechanisms [35,36]. As different from radical scavenging and reducing power assays, the order of phosphomolybdenum assay was *H. acutoconica* > *R. sanguinea* > *C. cylindracea > R. aurea* > *H. depilatum > L. deliciosus > N. erythropus > A. crocea*. The different order could be explained with the actions of non-phenolic antioxidants such as tocopherol and ascorbic acid in the assay. Thus, the assay could be considered as one total antioxidant capacity assay and not only phenolic, but also non-phenolic antioxidants could be effective role in the assay. As far as the literature could ascertain, many authors have reported the antioxidant properties for the tested mushrooms, such as *L. deliciosus* [7], *Cyclocybe cylindracea* [37] and *Amanita crocea* [25]. We have observed different results in these reports which could be justified based on the (equivalent or IC_50_ values) antioxidant assays.

### 3.3. Enzyme Inhibition Effects

The inhibition of key clinical enzymes have gained much momentum among the scientific as a therapeutic avenue for the management and prevention of global health problems including Alzheimer’s disease, diabetes mellitus and obesity. This fact is well known that the enzyme inhibition theory is based on the inhibition of key enzymes in the pathologies of these diseases. For example, cholinesterase is a key enzyme in the management of Alzheimer’s disease, which hydrolyzing acetylcholine in the synaptic gap. At this point, the inhibition of cholinesterase could increase the level of acetylcholine in the synaptic cleft and these effects may help to improve cognitive dysfunctions in Alzheimer patients [38]. In another example, the blood glucose level in diabetes mellitus patients was controlled by the inhibition of amylase and glucosidase, which are the main clinically relevant carbohydrate-hydrolyzing enzymes [39]. From these perspectives, a panoply of compounds have been produced as enzyme inhibitors in the pharmaceutical area. However, a number of studies has shown that the synthetic compounds exhibited undesirable side effects including gastrointestinal disturbances, toxicity [40,41]. Thus, there is a dire need to provide novel, safe and effective inhibitors instead of synthetic ones.

Taking the above into consideration, we evaluated the enzyme inhibitory properties of the mushroom extracts against some enzymes including cholinesterase, tyrosinase, amylase and glucosidase. The results are tabulated in Table 4. In cholinesterase inhibition assays, the best inhibitory effect was obtained by *C. cylindracea* (1.02 mg GALAE/g for AChE and 0.99 mg GALAE/g for BChE). In addition, five mushrooms were not active on BChE. In earlier studies, some mushroom species exhibited significant cholinesterase inhibitory effects. In particular, some mushroom metabolites and chemicals showed great potentials in terms of cholinesterase inhibition [41,42,43]. For example, Akata et al. [41,42,43], reported that the acetylcholinesterase inhibition abilities of some mushroom species varied from 0.83 to 0.97 mg GALAE/g extract. In addition, the acetylcholinesterase inhibition abilities were found to be 0.91 mg GALAE/g extract for *Hymenogaster aromaticus*, 1.02 mg GALAE/g extract for *Ramaria aurea* and 1.91 mg GALAE/g extract for *Rhizopogon luteolus* in an earlier study conducted by Zengin et al. [44]. In another study [1], two *Ganoderma* species (*G. applanatum* and *G. resinaceum*) displayed significant cholinesterase inhibition effects (AChE: 1.45 and 1.47 mg GALAE/g extract; BChE: 2.94 and 1.51 mg GALAE/g extract). Observed differences may be linked to the different myco-chemicals present in the extracts. Tyrosinase is a key enzyme in the synthesis of melanin, which is main pigment in skin and eyes. At this point, tyrosinase inhibition is useful to manage hyperpigmentation problems [45]. As can be seen from Table 4, tyrosinase inhibition effects were close in the tested mushroom extracts and the best ability was provided by *H. depilatum* with the value of 54.18 mg KAE/g. The weakest ability was detected in *R. sanguinea*. The observed tyrosinase inhibitory ability may be explained by the levels of phenolic compounds and this hypothesis was also corrected by correlation. In accordance with our results, some scientific studies indicated a positive correlation between tyrosinase inhibition ability and total phenolic content [46,47]. Also, the identified compounds, for instance *p*-hydroxybenzoic acid [48,49], *p*-coumaric acid [50], in the tested mushroom extracts were reported as significant tyrosinase inhibitors. Thus, the purported ability tend to be linked to the presence of these compounds. In addition, tyrosinase inhibition abilities were reported for several mushroom species in earlier studies, for example 4.43–12.86 mg KAE/g extract for two *Trametes* species [51]; 8.47–13.40 mg KAE/g extract for two *Ganoderma* [1] and 1.83–21.45 mg KAE/g extract for three medicinal mushroom [44]. The amylase inhibition effects for the mushroom extracts were close and the strongest effect was noted in *H. acutoconica* with value of 0.17-mmol ACAE/g, followed by *R. aurea* and *R. sanguinea*. Regarding glucosidase inhibition ability, two mushrooms (*A. crocea* and *H. depilatum*) were not active on glucosidase inhibition and the best ability was shown by *N. erythropus* with the value of 1.86-mmol ACAE/g. In the literature, some mushrooms exhibited remarkable anti-diabetic properties [52,53,54]. For instance, the amylase inhibitory effects of some mushroom species were found to be 0.28–0.40-mmol ACAE/g extract for three medicinal mushroom [44]; 0.16–0.22-mmol ACAE/g extract for six mushroom species [41,42,43]. Taken together, during the last century, diet or nutritional strategies are gaining much interest to control metabolic diseases and thus our findings could provide precious information for this purpose.

### 3.4. Determination of Mutagenicity

Table 5 shows the mean number of revertants/plate, the standard deviation after the treatments with the mushroom extracts, observed in *S. typhimurium* strains TA98, TA100, in the presence (+S9) and absence (−S9) of metabolic activation. According to the pre-screening of mutagenicity, it was determined that all mushroom extracts did not increase the revertant colony numbers at the test both with and without metabolic activation enzymes when compared with control plates (Table 5). We indicated above that if the revertant numbers of test plates are two-fold of spontaneous revertants, they can be considered as mutagen. Hence, our extracts did not reach to two-fold numbers of control plates. In other word, mushroom extracts tested in this study have not mutagenic potential.

### 3.5. Antimutagenic Evaluation

After determination of mutagenic potential of mushroom extracts, non-mutagenic extracts were assessed for their antimutagenicity against well-known mutagens. Table 6 shows the revertant colony numbers and inhibition rates of extracts.

*A. crocea* methanol extract can be considered as weak antimutagenic at all test doses (10,000, 5000 and 2500 µg/plate) against 4-NPDA for TA98 strain in the absence of S9 mix. The inhibition rates were determined as 15%, 19% and 22% (Table 6). The same extract revealed excellent antimutagenicity against 2-AF with a rate of 94% at a concentration of 10,000 µg/plate and 55% inhibition rate at a dose of 5000 µg/plate for TA98 strain after addition of metabolic activation enzymes. However, it was ineffective at the minimum dose (2500 µg/plate). For TA100 strain, *A. crocea* extracts did not reveal any antimutagenicity against SA at all test doses in the absence of S9 mix as in strain TA98. However, when this extract exposed to metabolic activation system, especially the highest dose of extract manifested strong antimutagenicity (83%) against 2-AA (Table 6). However, weak antimutagenic potential was observed at 5000 and 2500 µg/plate doses for TA100 strain. Both for two strains it can be said that metabolic activation system ameliorated the mutagenic effects of positive mutagens. When the *H. depilatum* methanol extract was evaluated, it was found that all tested concentrations had no activity against 4-NPDA for TA98 without S9 mix. By the addition of metabolic activation enzymes, this extract alleviated the mutagenic effect of 2-AF at a dose of 10,000 µg/plate with a rate of 93%, making the extract excellent antimutagenic (Table 6). The 5000 g/plate dose also revealed strong antimutagenicity with 41% inhibition rate. Except for 2500 g/plate concentration, other doses manifested moderate antimutagenic activity (32% and 32%, respectively) against SA for TA100 strain without S9 mix. After addition of S9 mix only 10,000 µg/plate dose revealed very strong inhibition (80%) against 2-AA, while other concentrations were considered to be weak action (Table 6). The potential antimutagenic action was not detected against 4-NPDA for TA98 strain at all test doses, although *C. cylindracea* extract associated with S9 showed strong activity against 2-AF with rates of 92% (10,000 µg/plate) and 40% (5000 µg/plate) (Table 6). The extract showed inhibition of revertants exceeding 27%, reaching 35% and 31% at three concentrations representing moderate antimutagenicity against SA for TA100 strain. Only 10,000 µg/plate dose manifested strong antimutagenicity against 2-AA with a rate of 84% with S9 mix for TA100 strain (Table 6); (L) *deliciosus* extract could not combat with the mutagenic effects of 4-NPDA without S9 for TA98, while metabolic activation enzymes induced the inhibition of revertants as 92% and 54% at doses of 10,000 and 5000 µg/plate for this strain against 2-AF (Table 6). Associated with SA, *Lactarius* extract was described as moderate antimutagenic for all test doses with ratios of 36%, 36% and 29%, respectively for TA100. Exposing to S9 mixture of plates affected the inhibition ratio only for 10,000 µg/plate dose with 58% inhibition ratio against 2-AA. Other doses had no antimutagenic capacity. When compared with other mushroom extracts, only *H. acutoconica* revealed strong antimutagenicity (45%) against 4-NPDA in the absence of S9 mix at a dose of 10,000 µg/plate for TA98. All tested concentrations showed more than 40% inhibition and concentrations in the range of 10,000–2500 μg/plate achieved 96%, 93% and 73% inhibition, respectively, making the extract a very strong antimutagen in the presence of metabolic activation system for TA98 (Table 6).

When *Hygrocybe* extracts were evaluated it demonstrated a potential for significant reduction in the numbers of revertants and it was found to be moderate antimutagenic at all test doses without S9 mix for TA100 against SA (38%, 34% and 36%, respectively). The highest inhibition rate (97%) was observed for 10,000 µg/plate dose of *Hygrocybe* and followed by 5000 µg/plate dose with a rate of 87% and 2500 µg/plate with 67% inhibition against 2-AA for TA100 in the presence of metabolic activation enzymes (Table 6). Furthermore, 97% ratio was the highest inhibition rates for whole study of Ames. *Neoboletus* extract was only effective against 2-AF for TA98 strain with a rate of 77% which was described as strong activity. Without S9 mix it had no capacity for combating with positive mutagens for TA98. However, this extract showed moderate antimutagenicity against SA at all doses with ratios of 35%, 31% and 28%, respectively. Except for 10,000 µg/plate dose, inhibition ratios decreased after addition of S9 and they assessed as weak activity. Only the highest dose revealed strong antimutagenicity against 2-AA with 50% inhibition rate (Table 6). Extract of *R. aurea* can be described as moderate antimutagenic against 4-NPDA for TA98 at 10,000 (34%) and 5000 (35%) µg/plate doses. It also determined as moderate antimutagenic for TA100 against SA at the same doses with 34% and 36% inhibitions. However, the supplement of metabolic activation mixture increased the inhibition ratios as 92% and 71% (very strong activity) for TA98 against 2-AF and extract strongly ameliorated the mutagenic effect of 2-AA with a ratio of 85% at a concentration of 10,000 µg/plate for TA100 (Table 6); (R) *sanguinea* extract manifested moderate antimutagenicity against 4-NPDA at 10,000 µg/plate dose, while it had very strong action against 2-AF with ratios of 94% and 78% in the presence of S9 mix for TA98. When this extract evaluated for TA100 strain, it induced the inhibitions greater than 25%, reaching 38%, 26% and 28%, respectively making them as moderate antimutagenic for all test doses. Only 10,000 µg/plate dose strongly alleviated the mutagenic actions of 2-AA with 85% inhibition in the presence of S9.

The results showed that there were no mutagenic properties of mushrooms tested, but they revealed significant antimutagenicity against well-known mutagens in the presence of metabolic activation enzymes. The best antimutagenicity against 2-AA was defined for *Hygrocybe acutoconica* extracts with a rate of 97% inhibition with S9 mix. This powerful action was followed *by Amanita crocea* and *Russula aurea* extracts with a ratio of 94% inhibition; *Hemileccinum depilatum* 93% inhibition and *Cyclocybe cylindracea* and *Lactarius deliciosus* 92% inhibition, respectively. It was observed that metabolic activation enzymes induced the antimutagenic action of the extracts in some mushrooms. It is the fact that the mushrooms exhibit a broad spectrum of pharmacological activities including antibacterial, antifungal, antiviral, cytotoxic, immunomodulating, anti-inflammatory, antioxidative, antiallergic, antidepressive, antihyperlipidemic, antidiabetic, digestive, hepatoprotective, neuroprotective, nephroprotective, osteoprotective and hypotensive activities [55]. Since the last decades ethnomedicinal and medicinal use of the mushrooms increased in the world wide [56]. In the previous studies although antimicrobial and antioxidant properties of mushrooms were well analyzed, their mutagenic and antimutagenic potentials were not well displayed. In a study conducted by Gruter et al. [57] *Craterellus* ethanol extract revealed powerful inhibition against 2-NF, AFB1, B(a)P and ICR-191. Furthermore, antimutagenic effects which are comparable with those of *Craterellus cornucopioides* were found in *Agaricus abruptibulbus*, *Agaricus bisporus*, *Cantharellus cibarius*, *Lactarius lilacinus*, *Lyophyllum connatum* and *Xerocomus chrysenteron*. Ham et al. [58] manifested that *Inonotus obliquus* extracts and their subfractions had a great potential of antimutagenicity (47% to 87% inhibition) in Ames test and this activity was attributed to that the 3β-hydroxy-lanosta-8, 24-dien-21-al and inotodiol components of *Inonotus obliquus*. Similarly Sugui et al. [59] studied Lentinula edodes extract for its inhibition on *N*-ethyl-*N*-nitrosourea (ENU) clastogenicity in vivo. The results showed that pretreatments with diets containing *L. edodes* lineages alleviated the micronucleated bone marrow polychromatic erythrocytes by N-ethyl-N-nitrosourea. In another study, the moderate (31–35%) antimutagenic action of polysaccharides extracted from *Agrocybe cylindracea* and *Phellinus igniarius* were revealed by Shon et al. [60]. Similarly, *Phellinus nigricans, Phellinus rimosus*, and *Phellinus wahlbergii* extracts were capable for combating with mutagens and they had strong antimutagenicity in Ames test Especially *P. rimosus* ethanol extract possessed excellent antimutagenic activity [61]. Morales et al. [62] exhibited that *Lactarius deliciosus* and *Boletus luteus* mushrooms were not mutagenic in the Salmonella/microsome test system. In our study eight mushroom extracts were not mutagenic and they also exhibited moderate to strong antimutagenicity against mutagens, so our results were consistent with authors’ results indicated above. In previous study of Mlinarič et al. [63], *Lactarius vellereus* extract was evaluated for its protective properties by Ames test and Comet assay and the results showed that it was highly curative against mutagens. In our study, *L. deliciosus* extract showed very strong activity against 2-AF in the presence of S9 mix. For the genus Russula previous studies focused on their cytotoxic and anti-proliferative effects showed that *R. cyanoxantha* revealed antitumor activity by the way of apoptosis [64]. Similarly, a lectin isolated from *Russula lepida* exhibited anti-proliferative activity toward Hep G2 cells and MCF-7 cells [65]. As a result of our study prescreening of the mutagenic and antimutagenic activity of *Russula aurea* and *Russula sanguinea* manifested that they were not only mutagenic, but also highly effective against 2-AF and 2-AA in the presence of metabolic activation enzymes.

Mushrooms produce many chemically diverse compounds with a broad spectrum of biologic activities. Because of the presence of some useful compounds such as polysaccharides, β-glucans, sacchachitin, tyrosinase and other enzymes there are increasing interest to probe into the potential of mushroom [55]. In vitro assays, animal studies, and a few clinical trials tend to justify the traditional experience and suggest a great potential of medicinal mushrooms and isolated compounds for the prophylaxis and treatment of several diseases. In this study, mutagenic and antimutagenic potential of eight mushroom were presented. This study can be considered as the first report on mutagenic and antimutagenic activities of *Amanita crocea*, *Hemileccinum depilatum*, *Cyclocybe cylindracea*, *Hygrocybe acutoconica*, *Neoboletus erythropu*, *Russula aurea* and *Russula sanguinea*. In the light of promising results, further detailed studies are needed to explore the potential of medicinal fungi and to promote the development of nutraceutical/functional foods and/or medicines.

### 3.6. Unsupervised Multivariate Analysis

**Figure 1:**
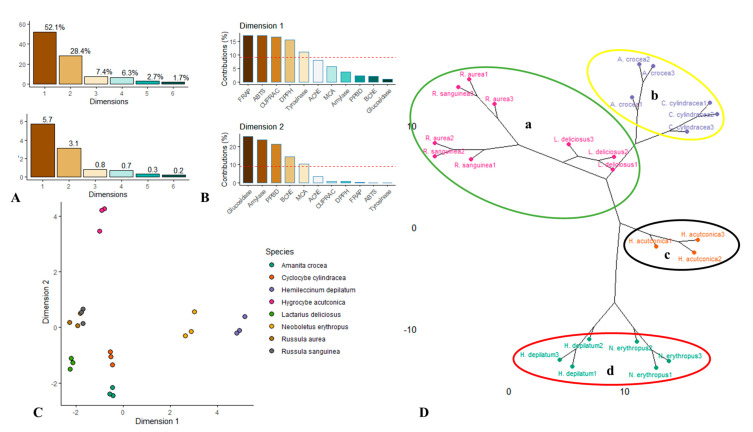
Principal component (PCA) and cluster analyses of bioactivities of mushroom species. (**A**) Percentage of explained variance and eigenvalue of dimensions of PCA; (**B**). contribution of bioactivities on the first two dimensions of PCA; (**C**) score plot of Dim 1 vs. Dim 2; (**D**) clustering of the species (a–d).

After the initial comparison of antioxidant and enzymatic inhibitory activities of the samples of the studied mushroom species through the univariate analysis, multivariate exploratory analyses, i.e., PCA and HCA were done with the intention of differentiating of those species and clustering them. In fact, PCA and HCA are often performed successively to detect homogenous groups and find the source of variation in the studied data. One of the advantage of these multivariate projections-based approaches is to display graphic results to easily visualize and interpret the results. Thus, first, unsupervised principal component analysis was employed to discriminate the mushroom species and the results are given in Figure 1. To identify the number of dimensions capturing the maximum of variability of the original, we have referred to “Kaiser rule” [66]. In this regard, two dimensions having an eigenvalue greater than one and contributing for more than 80% of the variability are retained (Figure 1A). The contribution of the bioactivity assays on the first two retained dimensions of PCA are reported in Figure 1B. As can be seen, the radical scavenging assays (ABTS and DPPH) and reducing power assays (FRAP, CUPRAC) had high contribution to the first dimension (52.1% of the total variance); implying that the species are separated along the first dimension according to their antioxidant activities. Likewise, the second dimension (28.4% of the total variance) had high loading for both antidiabetic assays (α-glucosidase and α-amylase) as well as phosphomolybdenum assay. The species are distinguished along the second dimension depending on their abilities to suppress glucosidase and amylase together with their antioxidant capacity. Figure 1C showed the projection of the samples on the 2D score plot of PCA (PC1 vs. PC2); there is a great variability between the species to the observation of the score plot. *N. erythropus* and *H. depilatum*, closed together, are separated from the other species, along the first dimension. In fact, relative to the other species, both these species showed the highest antioxidant properties. Furthermore, the second dimension separated *H. acutoconica*, *R. sanguinea* and *R. aurea* from *L. deliciosus*, *C. cylindracea* and *A. crocea.* In addition, *R. sanguinea*, *R. aurea* and especially *H. acutoconica* exhibited the strongest anti-amylase, anti-glucosidase activities and total antioxidant capacity.

In the next step, hierarchical clustering analysis was applied to highlight the different homogeneous clusters. The clustering analysis was done from the results of PCA taking into account the first two dimensions. Ward’s method and Euclidean were used as linkage algorithm and metric, respectively. The phylogenic map obtained revealed four main clusters (denoted as A, B, C and D). The cluster A comprised three species, i.e., *R. sanguinea*, *R. aurea* and especially *H. acutoconica;* the cluster B is represented by *A. crocea* and *C. cylindracea* species, the cluster C included *H. acutoconica* and the cluster D contained *N. erythropus* and *H. depilatum* species.

PCA and HCA allows the statistically significant discrimination of mushroom species according to their bioactivities. For two species including *N. erythropus* and *H. depilatum*, the significant antioxidant activities was obtained. Many publications have documented the presence of specific bioactive compounds like tocopherols, polysaccharides, ergosterol, ascorbic acid and phenolic as responsible for the antioxidant properties of various mushrooms species [67]. These compounds make mushrooms an alternative attractive source of food to deal with damage caused by the oxidation reaction of free radicals in the human body. On the other hand, *H. acutoconica* is found to possess good anti-amylase, anti-glucosidase activities. In fact, the anti-diabetic potential of several mushrooms is very well documented. For example, water extracts of wild edible mushrooms, i.e., *Clitocybe maxima*, *Stropharia rugoso-annulata*, *Craterellus cornucopioides*, *Catathelasma ventricosum*, *Laccaria amethystea* and *Catathelasma ventricosum* have been shown to have potent inhibition of alpha-glucosidase activity [68]. Similarly *G. frondosa* and *C. versicolor* have been reported to possess strong inhibitory effect on alpha-glucosidase and alpha-amylase activities, respectively [53].

To sum up, this was the first investigation highlighting the therapeutic value, i.e., the antioxidant potentiality and the potent antidiabetic activity of *N. erythropus*, *H. depilatum* and *H. acutoconica*.

## 4. Conclusions

Mushrooms displaying biologic activities have attracted much interest of both the pharmaceutical and food industries. In light of the data presented in this present study, the eight mushroom under investigations showed interesting biologic properties and chemical profiles; (H) *depilatum* and *N. erythropus* showed best radical quenching and reducing power abilities among the tested samples. In addition, different enzymatic inhibitory propensities were observed from the tested mushroom extracts. The mushroom samples showed significant anti-mutagenic potentials and none of them exhibited mutagenic effects. They alleviated significant mutagenic actions of 2-amino flouren and 2-amino anthracene by the inhibition ratios. Taking into consideration these facts, the mushroom species under investigation can be regarded as valuable sources of bioactive compounds to design novel ingredients. However, further experimental studies, both in vivo and/or bioavailability, are required to provide a more comprehensive therapeutic profile of these mushrooms.

## Figures and Tables

**Table 1:** Location of the mushroom tested and edibility properties.

Species	Habitat	Date	Edibility
*Amanita crocea* (Quél.) Singer	*Pinus* forest	June 2018	Edible
*Hemileccinum depilatum* (Redeuilh) Šutara	*Pinus* forest	September 2018	Edible
*Cyclocybe cylindracea* (DC.) Vizzini & Angelini	On *Populus* spp. stump	March 2018	Edible
*Lactarius deliciosus* (L.) Gray	*Pinus* forest	June 2018	Edible
*Hygrocybe acutoconica* (Clem.) Singer	Between meadows and grass	April 2018	Inedible
*Neoboletus erythropus* (Pers.) C. Hahn	*Pinus* forest	October 2018	Edible
*Russula aurea* Pers.	*Pinus* forest	June 2018	Edible
* Russula sanguinea * Fr.	*Pinus* forest	May 2018	Edible

**Table 2:** Total phenolic content and antioxidant properties of the mushroom species *.

Species	Total Phenolic Content (mg GAE/g)	DPPH (mg TE/g)	ABTS (mg TE/g)	CUPRAC (mg TE/g)	FRAP (mg TE/g)	Metal Chelating (mg EDTAE/g)	Phosphomolybdenum (mmol TE/g)
*Amanita crocea*	9.33 ± 0.06 ^e^	26.09 ± 0.17 ^c^	36.59 ± 2.53 ^d^	39.57 ± 1.19 ^d,e^	37.40 ± 0.78 ^c^	15.06 ± 0.02 ^a^	0.35 ± 0.05 ^d^
*Hemileccinum depilatum*	20.10 ± 0.46 ^a^	41.89 ± 1.05 ^a^	84.33 ± 0.72 ^a^	116.44 ± 7.30 ^a^	86.23 ± 3.22 ^a^	13.85 ± 0.52 ^a,b,c^	0.47 ± 0.02 ^c^
*Cyclocybe cylindracea*	10.53 ± 0.13 ^d^	20.53 ± 0.21 ^d^	41.49 ± 0.34 ^c^	44.49 ± 0.15 ^d^	31.69 ± 0.12 ^d^	14.91 ± 0.04 ^a^	0.61 ± 0.03 ^a^
*Lactarius deliciosus*	7.42 ± 0.08 ^f^	5.66 ± 0.04 ^g^	17.53 ± 0.78 ^g^	31.90 ± 0.37 ^f^	16.65 ± 0.39 ^g^	14.82 ± 0.08 ^a^	0.46 ± 0.03 ^c^
*Hygrocybe acutoconica*	10.49 ± 0.22 ^d^	14.60 ± 1.12 ^e^	36.18 ± 1.59 ^d^	56.31 ± 0.06 ^c^	37.44 ± 1.69 ^c^	13.44 ± 0.81 ^b,c^	0.81 ± 0.04 ^a^
*Neoboletus erythropus*	16.52 ± 0.22 ^b^	30.36 ± 3.16 ^b^	70.96 ± 0.45 ^b^	103.87 ± 0.78 ^b^	65.76 ± 0.23 ^b^	12.85 ± 0.87 ^c^	0.41 ± 0.03 ^c,d^
*Russula aurea*	11.36 ± 0.19 ^c^	10.11 ± 0.41 ^f^	25.35 ± 0.15 ^f^	29.36 ± 0.05 ^f^	21.06 ± 0.68 ^f^	14.87 ± 0.39 ^a^	0.58 ± 0.04 ^b^
*Russula sanguinea*	10.89 ± 0.15 ^e,d^	9.28 ± 0.12 ^f^	30.07 ± 0.26 ^e^	34.40 ± 0.20 ^f^	27.12 ± 0.33 ^e^	14.75 ± 0.23 ^a,b^	0.66 ± 0.04 ^b^

* Values reported as mean ± SD; GAE—gallic acid equivalent; TE—trolox equivalent; EDTAE—EDTA equivalent. Different superscripts indicate significant differences in the mushroom extracts (^a–g^, “^a^” indicates the highest content or activity; “^g^” indicates the lowest content or activity); (*p* < 0.05).

**Table 3:** Chemical composition of the tested mushroom extracts.

Compounds	Concentration of Compounds (µg/g)							
	1	2	3	4	5	6	7	8
*p*-Hydroxybenzoic acid	56.10	14.31	105.73	63.32	22.77	12.64	<9.77	<9.77
Cinnamic acid	539.38	<312.5	<312.5	<312.5	<312.5	<312.5	<312.5	<312.5
Protocatechuic acid	<9.77	<9.77	<9.77	<9.77	<9.77	<9.77	<9.77	<9.77
2,5-Dihydroxybenzoic acid	<9.77	<9.77	<9.77	<9.77	<9.77	<9.77	<9.77	<9.77
umbelliferon	<4.88	<4.88	<4.88	<4.88	<4.88	<4.88	<4.88	<4.88
*p*-Coumaric acid	2.81	2.67	6.98	<2.44	6.22	<2.44	<2.44	<2.44
o-Coumaric acid	<4.88	<4.88	<4.88	<4.88	<4.88	<4.88	<4.88	<4.88
Vanillic acid	<156.3	<156.3	<156.3	<156.3	<156.3	<156.3	<156.3	<156.3
Gallic acid	<39.1	<39.1	<39.1	<39.1	<39.1	<39.1	<39.1	<39.1
Esculetin	<19.5	<19.5	<19.5	<19.5	<19.5	<19.5	<19.5	<19.5
Caffeic acid	<9.77	<9.77	<9.77	<9.77	<9.77	<9.77	<9.77	<9.77
Hinic acid	36.40	19.4	26.81	14.17	14.60	8.50	32.48	23.32
Scopolamine	<19.5	<19.5	<19.5	<19.5	<19.5	<19.5	<19.5	<19.5
Ferulic acid	<9.77	<9.77	<9.77	<9.77	<9.77	<9.77	<9.77	<9.77
Sinapic acid	<78.1	<78.1	<78.1	<78.1	<78.1	<78.1	<78.1	<78.1
3,4-Dimethoxycinnamic acid	<78.1	<78.1	<78.1	<78.1	<78.1	<78.1	<78.1	<78.1
Sinapic acid	<39.1	<39.1	<39.1	<39.1	<39.1	<39.1	<39.1	<39.1
Daidzein	<19.5	<19.5	<19.5	<19.5	<19.5	<19.5	<19.5	<19.5
Genistein	<4.88	<4.88	<4.88	<4.88	<4.88	<4.88	<4.88	<4.88
Apigenin	<2.44	<2.44	<2.44	<2.44	<2.44	<2.44	<2.44	<2.44
Baicalin	<2.44	<2.44	<2.44	<2.44	<2.44	<2.44	<2.44	<2.44
Naringenin	<1.22	<1.22	<1.22	<1.22	<1.22	<1.22	<1.22	<1.22
Luteolin	<2.44	<2.44	<2.44	<2.44	<2.44	<2.44	<2.44	<2.44
kaempferol	<39.1	<39.1	<39.1	<39.1	<39.1	<39.1	<39.1	<39.1
Catechin	<39.1	<39.1	<39.1	<39.1	<39.1	<39.1	<39.1	<39.1
Epicatechin	<39.1	<39.1	<39.1	<39.1	<39.1	<39.1	<39.1	<39.1
Chrysoeriol	<0.61	<0.61	<0.61	<0.61	<0.61	<0.61	<0.61	<0.61
Quercetin	<312.5	<312.5	<312.5	<312.5	<312.5	<312.5	<312.5	<312.5
Isorhamnetin	<312.5	<312.5	<312.5	<312.5	<312.5	<312.5	<312.5	<312.5
Myricetin	<625	<625	<625	<625	<625	<625	<625	<625
Chlorogenic acid	<1.22	<1.22	6.81	5.49	<1.22	<1.22	<1.22	<1.22
Matairesinol	<4.88	<4.88	<4.88	<4.88	<4.88	7.68	<4.88	<4.88
Secoisolariciresinol	<9.77	<9.77	<9.77	<9.77	<9.77	<9.77	<9.77	<9.77
Vitexin	<2.44	<2.44	<2.44	<2.44	<2.44	<2.44	<2.44	<2.44
Apigenin-7-O-glc	<1.22	<1.22	<1.22	<1.22	<1.22	<1.22	<1.22	<1.22
Baicalin	<39.1	<39.1	<39.1	<39.1	<39.1	<39.1	<39.1	<39.1
Luteolin-7-O-glc	<1.22	<1.22	<1.22	<1.22	<1.22	<1.22	<1.22	<1.22
Quercitrin	<2.44	<2.44	<2.44	<2.44	<2.44	<2.44	<2.44	<2.44
Kaempferol 3-O-glucoside	<4.88	<4.88	<4.88	<4.88	<4.88	<4.88	<4.88	<4.88
Epigallocatechin gallate	<312.5	<312.5	<312.5	<312.5	<312.5	<312.5	<312.5	<312.5
Hyperoside	<4.88	<4.88	<4.88	<4.88	<4.88	<4.88	<4.88	<4.88
Quercetin 3-O-glucoside	<4.88	<4.88	<4.88	<4.88	<4.88	<4.88	<4.88	<4.88
Amentoflavone	<0.61	<0.61	<0.61	<0.61	<0.61	1.02	<0.61	<0.61
Apiin	<2.44	<2.44	<2.44	<2.44	<2.44	<2.44	<2.44	<2.44
Rutin	<4.88	<4.88	<4.88	<4.88	<4.88	<4.88	<4.88	<4.88

1—Amanita crocea; 2—Hemileccinum depilatum; 3—Cyclocybe cylindracea; 4—Lactarius deliciosus; 5—Hygrocybe acutoconica; 6—Neoboletus erythropus; 7—Russula aurea; 8—Russula sanguinea.

**Table 4:** Enzyme inhibitory properties of the tested mushroom extracts *.

Species	AChE (mg GALAE/g)	BChE (mg GALAE/g)	Tyrosinase (mg KAE/g)	Amylase (mmol ACAE/g)	Glucosidase (mmol ACAE/g)
*Amanita crocea*	0.90 ± 0.01 ^b^	0.80 ± 0.02 ^b^	49.79 ± 0.15 ^d,e^	0.08 ± 0.01 ^f,g^	NA
*Hemileccinum depilatum*	NA	NA	54.18 ± 0.17 ^a^	0.07 ± 0.01 ^g^	0.17 ± 0.07 ^c^
*Cyclocybe cylindracea*	1.02 ± 0.02 ^a^	0.99 ± 0.07 ^a^	53.24 ± 0.12 ^a,b^	0.09 ± 0.01 ^e,f^	0.34 ± 0.01 ^b^
*Lactarius deliciosus*	0.90 ± 0.01 ^b^	0.98 ± 0.05 ^a^	50.47 ± 0.46 ^c,d^	0.10 ± 0.01 ^c,d^	0.36 ± 0.01 ^b^
*Hygrocybe acutoconica*	0.60 ± 0.06 ^d^	NA	50.97 ± 0.45 ^c^	0.17 ± 0.01 ^a^	1.86 ± 0.01 ^a^
*Neoboletus erythropus*	0.95 ± 0.04 ^b^	NA	52.64 ± 0.16 ^b^	0.09 ± 0.01 ^d,e^	0.22 ± 0.04 ^c^
*Russula aurea*	0.93 ± 0.01 ^b^	NA	49.66 ± 0.59 ^d,e^	0.12 ± 0.01 ^b^	0.36 ± 0.01 ^b^
*Russula sanguinea*	0.78 ± 0.03 ^c^	NA	48.83 ± 0.30 ^e^	0.11 ± 0.01 ^b,c^	0.36 ± 0.01 ^b^

* Values reported as mean ± SD; GALAE—galantamine equivalent; KAE—kojic acid equivalent; ACAE—acarbose equivalent; NA—not active. Different superscripts indicate significant differences in the mushroom extracts (^a–g^, “^a^” indicates the highest activity; “^g^” indicates the lowest activity); (*p* < 0.05).

**Table 5:** Mutagenic properties of the tested mushrooms.

	Concentration µg/plate	TA 98		TA 100	
		S9 (−)	S9 (+)	S9 (−)	S9 (+)
Positive control		463 ± 42	2845 ± 109	1048 ± 131	4764 ± 129
Negative control	100 µL	32 ± 2	40 ± 3	139 ± 10	145 ± 6
Bacteria control	0	32 ± 1	36 ± 2	140 ± 15	151 ± 18
*Amanita crocea*	10,000	21 ± 1	29 ± 1	103 ± 3	138 ± 13
	5000	23 ± 4	34 ± 4	123 ± 6	147 ± 16
	2500	29 ± 4	36 ± 2	148 ± 4	152 ± 8
*Hemileccinum depilatum*	10,000	41 ± 3	41 ± 4	152 ± 13	163 ± 12
	5000	33 ± 3	40 ± 3	162 ± 11	170 ± 11
	2500	34 ± 1	32 ± 0	171 ± 7	159 ± 7
*Cyclocybe cylindracea*	10,000	37 ± 1	30 ± 2	172 ± 4	144 ± 8
	5000	31 ± 4	37 ± 2	139 ± 10	142 ± 10
	2500	36 ± 0	34 ± 3	151 ± 1	138 ± 4
*Lactarius deliciosus*	10,000	29 ± 1	40 ± 0	146 ± 4	155 ± 8
	5000	27 ± 1	28 ± 1	154 ± 12	146 ± 2
	2500	27 ± 3	37 ± 2	134 ± 12	138 ± 7
*Hygrocybe acutoconica*	10,000	43 ± 4	36 ± 3	147 ± 11	161 ± 14
	5000	30 ± 1	36 ± 7	130 ± 7	135 ± 11
	2500	31 ± 4	33 ± 1	151 ± 7	148 ± 9
*Neoboletus erythropus*	10,000	39 ± 3	30 ± 4	145 ± 8	129 ± 6
	5000	34 ± 5	43 ± 3	152 ± 13	135 ± 18
	2500	32 ± 5	41 ± 3	137 ± 1	144 ± 12
*Russula aurea*	10,000	28 ± 4	40 ± 5	135 ± 4	164 ± 9
	5000	31 ± 0	39 ± 1	145 ± 1	149 ± 14
	2500	34 ± 3	38 ± 2	132 ± 8	119 ± 12
*Russula sanguinea*	10,000	38 ± 4	37 ± 1	140 ± 3	128 ± 19
	5000	26 ± 2	29 ± 1	160 ± 5	162 ± 5
	2500	33 ± 3	31 ± 3	130 ± 6	143 ± 8

**Table 6:** Antimutagenic properties of the tested mushrooms.

	Concentration (µg/plate)	TA 98				TA 100			
		S9 (-)	% Inhibition	S9 (+)	% Inhibition	S9 (-)	% Inhibition	S9 (+)	% Inhibition
Negative Control	100 µL/plate	29 ± 2		41 ± 3		158 ± 8		170 ± 3	
Positive Control		667 ± 19	0	2796 ± 139	0	1730 ± 104	0	3631 ± 139	0
Bacteria Control	0	31 ± 4		41 ± 4		157 ± 17		175 ± 9	
*Amanita crocea*	10,000	569 ± 17	15	203 ± 16	94	1633 ± 34	6	773 ± 26	83
	5000	543 ± 20	19	1280 ± 25	55	1678 ± 28	3	3359 ± 49	8
	2500	524 ± 16	22	2457 ± 112	12	1407 ± 24	21	3519 ± 183	3
*Hemileccinum depilatum*	10,000	588 ± 32	12	237 ± 22	93	1225 ± 4	32	880 ± 46	80
	5000	553 ± 22	18	1679 ± 36	41	1233 ± 15	32	3382 ± 52	7
	2500	514 ± 25	24	2701 ± 126	3	1342 ± 36	25	3491 ± 103	4
*Cyclocybe cylindracea*	10,000	551 ± 25	18	254 ± 2	92	1185 ± 53	35	739 ± 31	84
	5000	560 ± 37	17	1697 ± 105	40	1241 ± 7	31	2904 ± 71	21
	2500	588 ± 42	12	2619 ± 39	6	1313 ± 34	27	3575 ± 151	2
*Lactarius deliciosus*	10,000	535 ± 11	21	260 ± 21	92	1166 ± 30	36	1617 ± 45	58
	5000	519 ± 10	23	1310 ± 118	54	1156 ± 57	36	3361 ± 61	8
	2500	580 ± 19	14	2760 ± 125	1	1281 ± 50	29	3312 ± 112	9
*Hygrocybe acutoconica*	10,000	379 ± 35	45	156 ± 19	96	1127 ± 14	38	279 ± 5	97
	5000	527 ± 16	22	242 ± 10	93	1188 ± 21	34	630 ± 54	87
	2500	565 ± 22	16	790 ± 46	73	1171 ± 4	36	1308 ± 102	67
*Neoboletus erythropus*	10,000	513 ± 20	24	676 ± 46	77	1186 ± 33	35	1900 ± 75	50
	5000	544 ± 25	19	2196 ± 102	22	1246 ± 14	31	3322 ± 114	9
	2500	571 ± 5	15	2594 ± 129	7	1292 ± 39	28	3527 ± 78	3
*Russula aurea*	10,000	449 ± 13	34	262 ± 16	92	1189 ± 51	34	694 ± 11	85
	5000	442 ± 6	35	847 ± 23	71	1171 ± 24	36	2306 ± 27	38
	2500	573 ± 11	15	2449 ± 131	13	1446 ± 49	18	2769 ± 46	25
*Russula sanguinea*	10,000	489 ± 18	28	213 ± 23	94	1138 ± 33	38	705 ± 38	85
	5000	461 ± 22	32	637 ± 26	78	1341 ± 16	26	2840 ± 44	23
	2500	591 ± 5	12	2025 ± 36	28	1287 ± 41	28	3386 ± 151	7

## Supplementary Materials

The following are available online at https://www.mdpi.com/2309-608X/6/3/166/s1.
